# Feature-specific predictive processing: What’s in a prediction error?

**DOI:** 10.1162/IMAG.a.1061

**Published:** 2025-12-18

**Authors:** David Richter, Cem Uran, Martin Vinck, Floris P. de Lange

**Affiliations:** Mind, Brain and Behavior Research Center (CIMCYC), University of Granada, Granada, Spain; Donders Institute for Brain, Cognition and Behaviour, Radboud University Nijmegen, Nijmegen, the Netherlands; Ernst Strüngmann Institute (ESI) for Neuroscience, Frankfurt am Main, Germany; Donders Centre for Neuroscience, Department of Neurophysics, Radboud University Nijmegen, Nijmegen, the Netherlands

**Keywords:** convolutional neural networks, deep neural networks, firing rates, fMRI, gamma oscillations, hierarchy, predictability, prediction error, predictive coding, predictive processing, visual cortex

## Abstract

Despite numerous studies reporting sensory prediction errors—a key component of predictive processing theories—the nature of the surprise represented in these errors remains largely unknown. Here, we highlight recent studies, which provide evidence that prediction errors, even in early sensory areas, may reflect high-level surprise, offering new insights into the role of predictive processing in the brain beyond classical accounts of redundancy reduction.

The brain has been described as a “prediction machine” that generates expectations about its environment to facilitate perception and decision-making (Friston, 2005; Rao & Ballard, 1999). Studies have demonstrated that neural responses are positively correlated to the amount of surprise that a stimulus elicits, both in the domain of sensory processing such as vision (Alink et al., 2010; den Ouden et al., 2010; Egner et al., 2010; Kaposvari et al., 2018; Kok et al., 2012; Meyer & Olson, 2011; Ramachandran et al., 2016; Richter et al., 2018) and audition (An et al., 2021; Gelens et al., 2024; Han et al., 2019; Lieder et al., 2013; Parras et al., 2017; Vidal et al., 2019), and beyond (Heilbron et al., 2022; Matsumoto et al., 2007; Oemisch et al., 2019); reviews (de Lange et al., 2018; Heilbron & Chait, 2018; Walsh et al., 2020). These studies conventionally compared neural responses between two conditions: predicted and surprising stimuli. In an experiment, these two categories can be well defined, for example, by presenting specific temporal sequences of stimuli that define predicted and surprising stimuli. Indeed, experiments often use simple artificial stimuli (e.g., gratings) and manipulate one specific stimulus feature (e.g., orientation) to create these two conditions (Furutachi et al., 2024). However, natural stimuli are complex constellations of many lower and higher-order features. Consequently, stimulus predictability naturally varies across many dimensions, which, we argue, is critical to investigate for charting the neurocomputational principles underlying perceptual inference. For example, if we move our eyes towards the location where we hear the honking sound of a car, we can strongly predict some features (e.g., specific rectilinear features and object size) while other features are completely unpredictable (e.g., the color of the car). In other words, a stimulus may be both predicted and surprising, depending on the specific features.

In our opinion, this poses a fundamental question: *What* does the visual system predict? Instead of focusing solely on the magnitude of prediction error responses, new research has attempted to expose the contents of prediction errors at different levels of the cortical hierarchy, which can elucidate the brain’s internal models and thereby constrain models of predictive processing. We refer to this new paradigm as *feature-specific predictive processing.* Using convolutional neural networks (CNN), which have successfully been used to study the encoding of natural images by the visual ventral stream (Yamins et al., 2014), recent studies have started to investigate this by decomposing sensory surprise into lower- and higher-level visual features.

First, Uran et al. (2022) explored how spatial predictability in natural images influences neural responses in macaque V1. Spatial predictability here referred to how well an image part could be predicted by its spatial surround, which was quantified using an inpainting algorithm. Then, they quantified the difference in CNN (Fig. 1A) activity patterns between the predicted and actual image patch, at low (early layers of the CNN, tuned to simple features like orientation and contrast) and high (late layers of the CNN, tuned to more complex features, such as objects and complex textures) levels, to determine low- and high-level predictability. They then investigated how low- and high-level predictability modulated distinct aspects of neural activity (Fig. 1B). V1 firing rates were weakly associated with low-level predictability but were strongly (and negatively) correlated with high-level predictability.

**Fig. 1.**
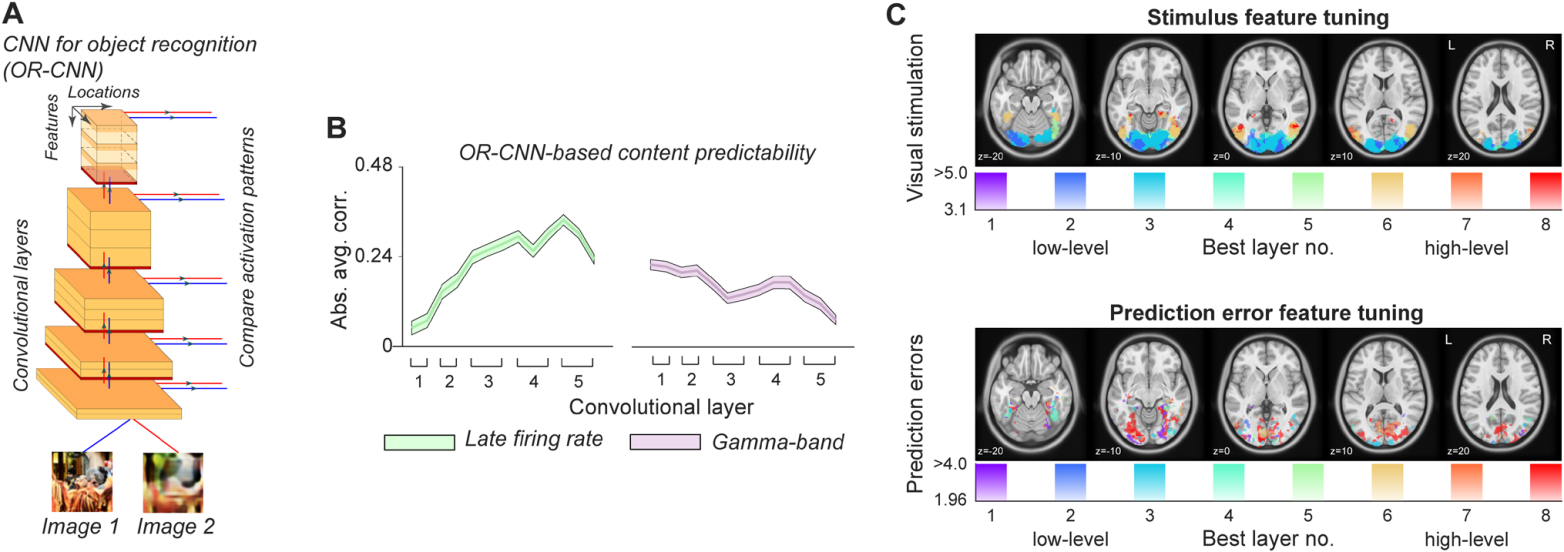
(A) Object classification CNNs (VGG-16 in Uran et al. (2022), and AlexNet in Richter et al. (2024, 2025)) were used to quantify stimulus predictability at different feature levels. (B) Correlation of macaque V1 neural activity with CNN predictability. Late multi-unit firing rates showed a significant increase in correlation across layers with image unpredictability, while γ-power showed a significant decrease. Figure adapted from Uran et al. (2022). (C) Representational similarity analysis showed a gradient from low-level to high-level visual feature processing along the ventral visual hierarchy during prediction-free contexts (top panel), with early visual cortex (EVC) aligning most with early CNN layers (cold colors) and higher visual areas more with late CNN layers (warm colors). In contrast, prediction error magnitudes increased with high-level visual feature surprise across the visual system, including in EVC (bottom panel). Figure adapted from Richter et al. (2024).

Second, Heilbron and de Lange (2025) used a similar analysis approach, investigating spatial predictability in mice. Results showed that visual cortical responses in mouse primary visual areas were again primarily modulated by high-level feature predictability. Moreover, this modulation by high-level predictability was the most pronounced in superficial layers of V1.

Third, Richter et al. used fMRI (Richter et al., 2024) and EEG (Richter et al., 2025) in humans to explore how temporal predictability affects the neural response in the visual ventral stream. Participants learned to expect a specific image given a cue. Following learning, participants were occasionally presented with a different, surprising, image, which could be differentially surprising in terms of low-level and high-level features. As expected, the ‘bottom-up’ stimulus feature tuning profile adhered to the classical visual hierarchy: V1 was tuned for simple features, reflected in early layers of the CNN, while higher-order visual regions were tuned to more complex features, reflected in late layers of the CNN (Fig. 1C, upper panel). Interestingly however, *all* visual areas from early occipital to higher-order ventral regions, including V1, were upregulated as a function of high-level, but not low-level, surprise (Fig. 1C, bottom panel; Richter et al., 2024). Moreover, using EEG, modulations by high-level surprise were observed relatively early, within 200 ms following stimulus onset (Richter et al., 2025).

We believe that despite differences in the type of predictions (spatial vs. temporal), populations studied (macaques, mice, humans), and recording methods (multi-unit, Neuropixel probes, fMRI, EEG), the above studies converge on a similar motif, namely that high-level visual surprise strongly drives neural activity, even in the earliest cortical visual areas, potentially as a result of feedback. While this convergence does not necessarily imply that these distinct manipulations and measurements isolate the exact same neurocomputational mechanism, it does suggest a shared operational principle, with related evidence showing that prediction errors in lower macaque face area ML inherit higher-level identity properties after learning (Schwiedrzik & Freiwald, 2017). Next, we discuss how these findings align with several models of predictive processing.

**(1) Hierarchical predictive coding (HPC).** HPC is a major model of cortical function that posits that feedback signals predictions about neural representations in the lower hierarchical level, while feedforward connections convey prediction errors arising from the comparison between top-down predictions and local representations (Rao & Ballard, 1999). Arguably, HPC thereby entails that higher areas signal prediction errors about higher-order features, while prediction errors about lower-order features are signaled in lower areas, because each area generates errors relative to predictions from the next hierarchical level. Thus, we argue that the findings of Uran et al. (2022), Heilbron and de Lange (2025), and Richter et al. (2024) that V1 activity increases for high- rather than low-level surprise may be difficult to reconcile with classic HPC implementations that depend on strict hierarchical error representation. In sum, HPC seems less consistent with the present evidence, given the non-hierarchical error representation evident by increased V1 activity for high-level surprise.

**(2) Feedback propagation of error signals.** An alternative to classic HPC is that error signals are generated in higher hierarchical levels and transmitted to lower levels via feedback connections (Heeger, 2017; also see: Whittington & Bogacz, 2019), where they lead to increased neural activity. V1 receives feedback from many higher-order sensory areas, allowing a direct modulation by top-down signals from many areas (Vezoli et al., 2021). In this scenario, feedback could serve to increase attention (Alink & Blank, 2021), update synaptic weights, aid in learning and novelty detection (Doron et al., 2020), and recruit additional neural resources when surprising events are encountered. This class of models would predict that V1 activity increases for high-level surprise, which we believe is consistent with the observations of the studies summarized above (Heilbron & de Lange, 2025; Richter et al., 2024; Uran et al., 2022). In this case, error signals may act as a scalar surprise signal that gain-modulates V1 responses, rather than subtract specific predictions (Furutachi et al., 2024). We believe that this account provides a parsimonious explanation of the combined findings (Heilbron & de Lange, 2025; Richter et al., 2024, 2025; Uran et al., 2022), as it naturally accounts for V1 upregulations by high-level surprise via feedback. This account is also compatible with ‘opposing process theory’, as proposed by Press et al. (2020), particularly with the feedback-based upweighting of surprising events, following the initial identification of the stimulus.

**(3) V1 as a comparator circuit for higher-level features.** Another possibility, which we consider to be in line with these recent findings, is that low-level areas like V1 may act as a comparator circuit for higher-order features. Local features can provide evidence for objects, and object recognition can be approximated by globally averaging over many local evidence filters (Farahat et al., 2023). For example, a local patch of yellow in a V1 receptive field provides some evidence for a banana, a sun, or an autumn leave, and if feedback into V1 signals the prediction of a blueberry, a higher-order prediction error could result when a local feature is not compatible with the high-level prediction. Thereby, this account explains how high-level error information is reflected in V1, but in contrast to (2) entails a prediction, instead of a scalar-like error signal being relayed via feedback.

**(4) Dendritic HPC.** Interestingly, the study of Uran et al. (2022) showed that while high-level predictability modulated V1 firing rates, low-level prediction errors primarily affected 30–80 Hz oscillatory, narrow-band (not broadband) gamma synchronization within V1. We argue that the dissociation between firing rates and gamma-synchronization fits well within the recently proposed dendritic HPC (dHPC) model (Aizenbud et al., 2025; Mikulasch et al., 2023; Vinck et al., 2025). The dHPC model implements a *mathematically equivalent algorithm* compared to classic HPC; however, dHPC maps the variables to different neural elements (Aizenbud et al., 2025; Mikulasch et al., 2023; Vinck et al., 2025). In dHPC, the basal dendritic compartment encodes feedforward prediction errors, implemented as interaction between excitatory feedforward inputs and local lateral inhibition. The apical compartment encodes the error of representations in level *k* with respect to predictions from level *k+1,* implemented as difference between top-down excitatory inputs and membrane leak currents. Importantly, dHPC entails that low-level predictability gives rise to a tighter balance of excitation and inhibition state, which is known to promote oscillatory gamma-synchronization (Denève & Machens, 2016; Vinck et al., 2025). Thus, we propose that V1 gamma-synchronization reflects the extent to which superficial-layer V1 representations can predict afferent excitatory inputs. The increase in V1 firing rates with higher-order predictability, on the other hand, should reflect the influence of prediction errors on the apical dendritic compartment, for example, via plateau potentials. Thereby, dHPC provides a plausible account of the combined findings (Heilbron & de Lange, 2025; Richter et al., 2024, 2025; Uran et al., 2022), linking low-level predictability to narrow-band gamma synchronization and high-level feedback to increased V1 firing rates.

Regardless of these different models, it is important that anatomical findings are in line with the notion that V1 can encode prediction errors about high-level features: V1 receives feedback from many higher-order areas (Vezoli et al., 2021), such that feedback concerning high-level features and error signals may reach it via direct projections. The fact that V1 receives feedback from many hierarchical levels, rather than only from the next level, as assumed in some predictive coding models, in our opinion provides a plausible anatomical basis for the finding that V1 activity signals mismatch signals about high-level features.

Does this feature-specific predictive processing strategy make sense from a behavioral standpoint? As the brain may be a “predictive machine” that continuously refines its internal models, a key question is: What are these predictions about? In principle, the brain could predict across all hierarchical levels, including fine-grained details of its sensory input, down to the level of pixels—as classic HPC models may suggest. However, higher-level features are likely more behaviorally relevant and may generally be easier to predict than lower-level features (e.g., rain vs individual drops of rain). Indeed, spatiotemporal prediction tasks like predicting future frames can be learned more effectively at the level of higher-order features rather than at the pixel level (Luc et al., 2017), and high-level predictions may be particularly effective for self-supervised learning of visual representations (Heilbron & de Lange, 2025). Finally, high-level stimulus predictability shows stronger correlations with human perceptual similarity judgements and salience maps (Uran et al., 2022). Thus, learning and behavior may be primarily guided by high-level predictability, and therefore we argue that perhaps the brain’s “prediction game” focuses on high-level, not low-level features in its sensory input.

Finally, considerable evidence supports that surprise increases sensory responses (reviews: de Lange et al., 2018; Heilbron & Chait, 2018; Walsh et al., 2020), yet findings are mixed with other studies showing limited or no evidence for such surprise scaling (Den Ouden et al., 2023, 2025; Solomon et al., 2021; Vinken et al., 2018) (review: Feuerriegel et al., 2021). While further work is required to delineate under which circumstances surprise may fail to scale sensory responses (e.g., differences in recording methods and paradigms), the hypothesized emphasis on high-level predictability may be part of the explanation. Contrasts between expected and unexpected inputs with limited variance in high-level features may yield little evidence for sensory responses being driven by surprise, especially when predictions are task-irrelevant and attention is drawn away from the predictable input (Richter & de Lange, 2019). Accordingly, decomposing surprise across levels of abstraction may increase sensitivity by aligning analyses with the features that may be particularly predicted by the sensory brain.

Together, these studies demonstrate what kind of predictions the brain makes in richer, more naturalistic, environments. Future challenges include: (1) extending feature-specific prediction error analyses to other sensory modalities, especially audition; (2) charting boundary conditions under which the predominant source of prediction may shift or span across levels of abstraction; (3) determining potential contributions of cortical arousal and pupil dilation associated with high-level surprise to early cortical responses; and (4) better integrating relevant biological implementation principles (e.g. dendritic HPC) with algorithmic and computational goals to generate a unified theory of predictive processing that spans across all of Marr’s levels of analysis.

## References

[IMAG.a.1061-b1] Aizenbud, I., Audette, N., Auksztulewicz, R., Basiński, K., Bastos, A. M., Canales-Johnson, A., Choi, H., Clopath, C., Cohen, U., Costa, R. P., Filippo, R. D., Doronin, R., Errington, S. P., Gavornik, J. P., Gillon, C. J., Hamm, J. P., Hertäg, L., Kennedy, H., Kumar, S., … Xiong, Y. S. (2025). Neural mechanisms of predictive processing: A collaborative community experiment through the OpenScope program. arXiv. 10.1534/g3.114.015966

[IMAG.a.1061-b2] Alink, A., & Blank, H. (2021). Can expectation suppression be explained by reduced attention to predictable stimuli? NeuroImage, 231, 117824. 10.1016/j.neuroimage.2021.117824 33549756

[IMAG.a.1061-b3] Alink, A., Schwiedrzik, C. M., Kohler, A., Singer, W., & Muckli, L. (2010). Stimulus predictability reduces responses in primary visual cortex. Journal of Neuroscience, 30(8), 2960–2966. 10.1523/JNEUROSCI.3730-10.2010 20181593 PMC6633950

[IMAG.a.1061-b4] An, H., Ho Kei, S., Auksztulewicz, R., & Schnupp, J. W. H. (2021). Do auditory mismatch responses differ between acoustic features? Frontiers in Human Neuroscience, 15, 613903. 10.3389/fnhum.2021.613903 33597853 PMC7882487

[IMAG.a.1061-b5] de Lange, F. P., Heilbron, M., & Kok, P. (2018). How do expectations shape perception? Trends in Cognitive Sciences, 22(9), 764–779. 10.1016/j.tics.2018.06.002 30122170

[IMAG.a.1061-b6] Den Ouden, C., Kashyap, M., Kikkawa, M., & Feuerriegel, D. (2025). Limited evidence for probabilistic cueing effects on grating-evoked event-related potentials and orientation decoding performance. Psychophysiology, 62(5), e70076. 10.1101/2024.05.26.595980 40391524 PMC12090177

[IMAG.a.1061-b7] Den Ouden, C., Zhou, A., Mepani, V., Kovács, G., Vogels, R., & Feuerriegel, D. (2023). Stimulus expectations do not modulate visual event-related potentials in probabilistic cueing designs. NeuroImage, 280, 120347. 10.1016/j.neuroimage.2023.120347 37648120

[IMAG.a.1061-b8] den Ouden, H. E. M., Daunizeau, J., Roiser, J., Friston, K. J., & Stephan, K. E. (2010). Striatal prediction error modulates cortical coupling. Journal of Neuroscience, 30(9), 3210–3219. 10.1523/JNEUROSCI.4458-09.2010 20203180 PMC3044875

[IMAG.a.1061-b9] Denève, S., & Machens, C. K. (2016). Efficient codes and balanced networks. Nature Neuroscience, 19(3), 375–382. 10.1038/nn.4243 26906504

[IMAG.a.1061-b10] Doron, G., Shin, J. N., Takahashi, N., Drüke, M., Bocklisch, C., Skenderi, S., De Mont, L., Toumazou, M., Ledderose, J., Brecht, M., Naud, R., & Larkum, M. E. (2020). Perirhinal input to neocortical layer 1 controls learning. Science, 370(6523), eaaz3136. 10.1126/science.aaz3136 33335033 PMC7612443

[IMAG.a.1061-b11] Egner, T., Monti, J. M., & Summerfield, C. (2010). Expectation and surprise determine neural population responses in the ventral visual stream. Journal of Neuroscience, 30(49), 16601–16608. 10.1523/JNEUROSCI.2770-10.2010 21147999 PMC3975573

[IMAG.a.1061-b12] Farahat, A., Effenberger, F., & Vinck, M. (2023). A novel feature-scrambling approach reveals the capacity of convolutional neural networks to learn spatial relations. Neural Networks, 167, 400–414. 10.1016/j.neunet.2023.08.021 37673027 PMC7616855

[IMAG.a.1061-b13] Feuerriegel, D., Vogels, R., & Kovács, G. (2021). Evaluating the evidence for expectation suppression in the visual system. Neuroscience & Biobehavioral Reviews, 126, 368–381. 10.1016/j.neubiorev.2021.04.002 33836212

[IMAG.a.1061-b14] Friston, K. (2005). A theory of cortical responses. Philosophical Transactions of the Royal Society B: Biological Sciences, 360(1456), 815–836. 10.1098/rstb.2005.1622 PMC1569488 15937014

[IMAG.a.1061-b15] Furutachi, S., Franklin, A. D., Aldea, A. M., Mrsic-Flogel, T. D., & Hofer, S. B. (2024). Cooperative thalamocortical circuit mechanism for sensory prediction errors. Nature, 633(8029), 398–406. 10.1038/s41586-024-07851-w 39198646 PMC11390482

[IMAG.a.1061-b16] Gelens, F., Äijälä, J., Roberts, L., Komatsu, M., Uran, C., Jensen, M. A., Miller, K. J., Ince, R. A. A., Garagnani, M., Vinck, M., & Canales-Johnson, A. (2024). Distributed representations of prediction error signals across the cortical hierarchy are synergistic. Nature Communications, 15(1), 3941. 10.1038/s41467-024-48329-7 PMC11087548 38729937

[IMAG.a.1061-b17] Han, B., Mostert, P., & de Lange, F. P. (2019). Predictable tones elicit stimulus-specific suppression of evoked activity in auditory cortex. NeuroImage, 200, 242–249. 10.1016/j.neuroimage.2019.06.033 31229656

[IMAG.a.1061-b18] Heeger, D. J. (2017). Theory of cortical function. Proceedings of the National Academy of Sciences, 114(8), 1773–1782. 10.1073/pnas.1619788114 PMC5338385 28167793

[IMAG.a.1061-b19] Heilbron, M., Armeni, K., Schoffelen, J.-M., Hagoort, P., & De Lange, F. P. (2022). A hierarchy of linguistic predictions during natural language comprehension. Proceedings of the National Academy of Sciences, 119(32), e2201968119. 10.1073/pnas.2201968119 PMC9371745 35921434

[IMAG.a.1061-b20] Heilbron, M., & Chait, M. (2018). Great expectations: Is there evidence for predictive coding in auditory cortex? Neuroscience, 389, 54–73. 10.1016/j.neuroscience.2017.07.061 28782642

[IMAG.a.1061-b21] Heilbron, M., & de Lange, F. P. (2025). Higher-level spatial prediction in natural vision across mouse visual cortex. bioRxiv. 10.1101/2025.05.15.654212 PMC12829946 41557751

[IMAG.a.1061-b22] Kaposvari, P., Kumar, S., & Vogels, R. (2018). Statistical learning signals in macaque inferior temporal cortex. Cerebral Cortex, 28(1), 250–266. 10.1093/cercor/bhw374 27909007

[IMAG.a.1061-b23] Kok, P., Jehee, J. F. M., & de Lange, F. P. (2012). Less is more: Expectation sharpens representations in the primary visual cortex. Neuron, 75(2), 265–270. 10.1016/j.neuron.2012.04.034 22841311

[IMAG.a.1061-b24] Lieder, F., Stephan, K. E., Daunizeau, J., Garrido, M. I., & Friston, K. J. (2013). A neurocomputational model of the mismatch negativity. PLoS Computational Biology, 9(11), e1003288. 10.1371/journal.pcbi.1003288 24244118 PMC3820518

[IMAG.a.1061-b25] Luc, P., Neverova, N., Couprie, C., Verbeek, J., & LeCun, Y. (2017). Predicting deeper into the future of semantic segmentation (No. arXiv:1703.07684). arXiv. http://arxiv.org/abs/1703.07684

[IMAG.a.1061-b26] Matsumoto, M., Matsumoto, K., Abe, H., & Tanaka, K. (2007). Medial prefrontal cell activity signaling prediction errors of action values. Nature Neuroscience, 10(5), 647–656. 10.1038/nn1890 17450137

[IMAG.a.1061-b27] Meyer, T., & Olson, C. R. (2011). Statistical learning of visual transitions in monkey inferotemporal cortex. Proceedings of the National Academy of Sciences, 108(48), 19401–19406. 10.1073/pnas.1112895108 PMC3228439 22084090

[IMAG.a.1061-b28] Mikulasch, F. A., Rudelt, L., Wibral, M., & Priesemann, V. (2023). Where is the error? Hierarchical predictive coding through dendritic error computation. Trends in Neurosciences, 46(1), 45–59. 10.1016/j.tins.2022.09.007 36577388

[IMAG.a.1061-b29] Oemisch, M., Westendorff, S., Azimi, M., Hassani, S. A., Ardid, S., Tiesinga, P., & Womelsdorf, T. (2019). Feature-specific prediction errors and surprise across macaque fronto-striatal circuits. Nature Communications, 10(1), 176. 10.1038/s41467-018-08184-9 PMC6329800 30635579

[IMAG.a.1061-b30] Parras, G. G., Nieto-Diego, J., Carbajal, G. V., Valdés-Baizabal, C., Escera, C., & Malmierca, M. S. (2017). Neurons along the auditory pathway exhibit a hierarchical organization of prediction error. Nature Communications, 8(1), 2148. 10.1038/s41467-017-02038-6 PMC5732270 29247159

[IMAG.a.1061-b31] Press, C., Kok, P., & Yon, D. (2020). The perceptual prediction paradox. Trends in Cognitive Sciences, 24(1), 13–24. 10.1016/j.tics.2019.11.003 31787500

[IMAG.a.1061-b32] Ramachandran, S., Meyer, T., & Olson, C. R. (2016). Prediction suppression in monkey inferotemporal cortex depends on the conditional probability between images. Journal of Neurophysiology, 115(1), 355–362. 10.1152/jn.00091.2015 26581864 PMC4760508

[IMAG.a.1061-b33] Rao, R. P. N., & Ballard, D. H. (1999). Predictive coding in the visual cortex: A functional interpretation of some extra-classical receptive-field effects. Nature Neuroscience, 2(1), 79–87. 10.1038/4580 10195184

[IMAG.a.1061-b34] Richter, D., & de Lange, F. P. (2019). Statistical learning attenuates visual activity only for attended stimuli. eLife, 8, e47869. 10.7554/eLife.47869 31442202 PMC6731093

[IMAG.a.1061-b35] Richter, D., Ekman, M., & de Lange, F. P. (2018). Suppressed sensory response to predictable object stimuli throughout the ventral visual stream. The Journal of Neuroscience, 38(34), 7452–7461. 10.1523/JNEUROSCI.3421-17.2018 30030402 PMC6596138

[IMAG.a.1061-b36] Richter, D., Kietzmann, T. C., & De Lange, F. P. (2024). High-level visual prediction errors in early visual cortex. PLoS Biology, 22(11), e3002829. 10.1371/journal.pbio.3002829 39527555 PMC11554119

[IMAG.a.1061-b37] Richter, D., Pena, P., & Ruz, M. (2025). Rapid computation of high-level visual surprise. iScience, 28(12), 114121. 10.1101/2025.06.20.660166 PMC12719766 41438059

[IMAG.a.1061-b38] Schwiedrzik, C. M., & Freiwald, W. A. (2017). High-level prediction signals in a low-level area of the Macaque face-processing hierarchy. Neuron, 96(1), 89-97.e4. 10.1016/j.neuron.2017.09.007 28957679 PMC5757317

[IMAG.a.1061-b39] Solomon, S. S., Tang, H., Sussman, E., & Kohn, A. (2021). Limited evidence for sensory prediction error responses in visual cortex of macaques and humans. Cerebral Cortex, 31(6), 3136–3152. 10.1093/cercor/bhab014 33683317 PMC8599921

[IMAG.a.1061-b40] Uran, C., Peter, A., Lazar, A., Barnes, W., Klon-Lipok, J., Shapcott, K. A., Roese, R., Fries, P., Singer, W., & Vinck, M. (2022). Predictive coding of natural images by V1 firing rates and rhythmic synchronization. Neuron, 110(7), 1240-1257.e8. 10.1016/j.neuron.2022.01.002 35120628 PMC8992798

[IMAG.a.1061-b41] Vezoli, J., Magrou, L., Goebel, R., Wang, X.-J., Knoblauch, K., Vinck, M., & Kennedy, H. (2021). Cortical hierarchy, dual counterstream architecture and the importance of top-down generative networks. NeuroImage, 225, 117479. 10.1016/j.neuroimage.2020.117479 33099005 PMC8244994

[IMAG.a.1061-b42] Vidal, Y., Brusini, P., Bonfieni, M., Mehler, J., & Bekinschtein, T. A. (2019). Neural signal to violations of abstract rules using speech-like stimuli. Eneuro, 6(5), ENEURO.0128-19.2019. 10.1523/ENEURO.0128-19.2019 31551251 PMC6787344

[IMAG.a.1061-b43] Vinck, M., Uran, C., Dowdall, J. R., Rummell, B., & Canales-Johnson, A. (2025). Large-scale interactions in predictive processing: Oscillatory versus transient dynamics. Trends in Cognitive Sciences, 29(2), 133–148. 10.1016/j.tics.2024.09.013 39424521 PMC7616854

[IMAG.a.1061-b44] Vinken, K., Op De Beeck, H. P., & Vogels, R. (2018). Face repetition probability does not affect repetition suppression in macaque inferotemporal cortex. The Journal of Neuroscience, 38(34), 7492–7504. 10.1523/JNEUROSCI.0462-18.2018 30030399 PMC6596142

[IMAG.a.1061-b45] Walsh, K. S., McGovern, D. P., Clark, A., & O’Connell, R. G. (2020). Evaluating the neurophysiological evidence for predictive processing as a model of perception. Annals of the New York Academy of Sciences, 1464(1), 242–268. 10.1111/nyas.14321 32147856 PMC7187369

[IMAG.a.1061-b46] Whittington, J. C. R., & Bogacz, R. (2019). Theories of error back-propagation in the brain. Trends in Cognitive Sciences, 23(3), 235–250. 10.1016/j.tics.2018.12.005 30704969 PMC6382460

[IMAG.a.1061-b47] Yamins, D. L. K., Hong, H., Cadieu, C. F., Solomon, E. A., Seibert, D., & DiCarlo, J. J. (2014). Performance-optimized hierarchical models predict neural responses in higher visual cortex. Proceedings of the National Academy of Sciences, 111(23), 8619–8624. 10.1073/pnas.1403112111 PMC4060707 24812127

